# A human PET study of [^11^C]HMS011, a potential radioligand for AMPA receptors

**DOI:** 10.1186/s13550-017-0313-0

**Published:** 2017-08-16

**Authors:** Keisuke Takahata, Yasuyuki Kimura, Chie Seki, Masaki Tokunaga, Masanori Ichise, Kazunori Kawamura, Maiko Ono, Soichiro Kitamura, Manabu Kubota, Sho Moriguchi, Tatsuya Ishii, Yuhei Takado, Fumitoshi Niwa, Hironobu Endo, Tomohisa Nagashima, Yoko Ikoma, Ming-Rong Zhang, Tetsuya Suhara, Makoto Higuchi

**Affiliations:** 1 Department of Functional Brain Imaging Research, National Institute of Radiological Sciences, National Institutes for Quantum and Radiological Science and Technology, 4-9-1 Anagawa, Inage-ku, Chiba, 263-8555 Chiba, Japan; 2 Department of Neuropsychiatry, Keio University School of Medicine, 35 Shinanomachi, Shinjuku-ku, 160-8582 Tokyo Japan; 3 Department of Clinical and Experimental Neuroimaging, Center for Development of Advanced Medicine for Dementia, National Center for Geriatrics and Gerontology, 7-430 Morioka-cho, Obu, 474-8511 Aichi Japan; 4 Department of Radiopharmaceuticals Development, National Institute of Radiological Sciences, National Institutes for Quantum and Radiological Science and Technology, 4-9-1 Anagawa, Inage-ku, Chiba, 263-8555 Chiba, Japan; 5 Research Imaging Centre, Centre for Addiction and Mental Health, 250 College Street, Toronto, M5T 1R8 ON Canada; 6 Department of Neurology, Kyoto Prefectural University of Medicine, 465 Kajii-cho, Hirokoji Agaru, Kawaramachi-dori, Kamigyo-ku, Kyoto, 602-8566 Kyoto, Japan; 7 Division of Neurology, Kobe University Graduate School of Medicine, 7-5-1, Kusunoki-cho, Chuo-ku, Kobe, 650-0017 Hyogo Japan; 8 Department of Molecular Imaging and Theranostics, National Institute of Radiological Sciences, National Institutes for Quantum and Radiological Science and Technology, 4-9-1 Anagawa, Inage-ku, Chiba, Chiba, 263-8555 Japan

**Keywords:** PET, Perampanel, AMPA, [^11^C]HMS011, Interspecies differences

## Abstract

**Background:**

α-Amino-3-hydroxy-5-methyl-4-isoxazole propionate (AMPA) receptor is a primary mediator of fast glutamatergic excitatory signaling in the brain and has been implicated in diverse neuropsychiatric diseases. We recently developed a novel positron emission tomography (PET) ligand, 2-(1-(3-([^11^C]methylamino)phenyl)-2-oxo-5-(pyrimidin-2-yl)-1,2-dihydropyridin-3-yl) benzonitrile ([^11^C]HMS011). This compound is a radiolabelled derivative of perampanel, an antiepileptic drug acting on AMPA receptors, and was demonstrated to have promising in vivo properties in the rat and monkey brains. In the current study, we performed a human PET study using [^11^C]HMS011 to evaluate its safety and kinetics.

Four healthy male subjects underwent a 120-min PET scan after injection of [^11^C]HMS011. Arterial blood sampling and metabolite analysis were performed to obtain parent input functions for three of the subjects using high-performance liquid chromatography. Regional distribution volumes (*V*_T_s) were calculated based on kinetic models with and without considering radiometabolite in the brain. The binding was also quantified using a reference tissue model with white matter as reference.

**Results:**

Brain uptake of [^11^C]HMS011 was observed quickly after the injection, followed by a rapid clearance. Three hydrophilic and one lipophilic radiometabolites appeared in the plasma, with notable individual variability. The kinetics in the brain with apparent radioactivity retention suggested that the lipophilic radiometabolite could enter the brain. A dual-input graphical model, an analytical model designed in consideration of a radiometabolite entering the brain, well described the kinetics of [^11^C]HMS011. A reference tissue model showed small radioligand binding potential (BP*_ND_) values in the cortical regions (BP*_ND_ = 0–0.15). These data suggested specific binding component of [^11^C]HMS011 in the brain.

**Conclusions:**

Kinetic analyses support some specific binding of [^11^C]HMS011 in the human cortex. However, this ligand may not be suitable for practical AMPA receptor PET imaging due to the small dynamic range and metabolite in the brain.

**Electronic supplementary material:**

The online version of this article (doi:10.1186/s13550-017-0313-0) contains supplementary material, which is available to authorized users.

## Background

Glutamate is the major excitatory neurotransmitter in the central nervous system. Among glutamatergic receptors, α-amino-3-hydroxy-5-methyl-4-isoxazole propionate (AMPA) receptors are largely located in the postsynaptic membrane in the cerebral cortex [1] and mediate fast excitatory signaling within the central nervous system. Animal and human studies have also implicated AMPA receptors in a wide range of brain disorders, including epilepsy [2], amyotrophic lateral sclerosis [3], mood disorders [4], and schizophrenia [5]. Therefore, a non-invasive method to visualize the density and distribution of AMPA receptors in the human brain would be a critical step for understanding the mechanisms underlying these neuropsychiatric disorders.

While considerable efforts have been made to develop PET ligands for other glutamate receptors such as the *N*-methyl-d-aspartate (NMDA) receptor family, AMPA receptor imaging has remained largely unexplored. Although a prototypical competitive AMPA receptor antagonist, 2,3-dihydroxy-6-nitro-7-sulfamoyl-benzo(f)quinoxaline (NBQX), demonstrated significant anticonvulsant effects [6], its clinical use has been prevented by its neurotoxicity and poor penetration of the blood-brain barrier [7].

Noncompetitive AMPA antagonists suggested to be more beneficial than competitive AMPA antagonists in the treatment of seizure, as their effectiveness is unaffected by high endogenous glutamate concentrations associated with excessive excitation of neurons [6]. Among them, perampanel, 2-(2-oxo-1-phenyl-5-pyridin-2-yl-1,2-dihydropyridin-3-yl) benzonitrile, has a high selectivity for AMPA receptors and was found to be effective in the treatment of epilepsy in animal models [8] and humans [9, 10], leading to FDA approval as an adjunctive antiepileptic treatment in patients with uncontrolled partial-onset seizures. As perampanel showed good brain uptake and adequate affinity and selectivity for AMPA receptors [8], its derivatives could be suitable PET probes for in vivo AMPA receptor imaging.

Based on these findings, we developed a derivative of perampanel, 2-(1-(3-([^11^C]methylamino)phenyl)-2-oxo-5-(pyrimidin-2-yl)-1,2-dihydropyridin-3-yl) benzonitrile ([^11^C]HMS011), as a PET ligand for AMPA receptors. In our previous study, [^11^C]HMS011 autoradiography showed specific binding to the neocortex and hippocampus of the rat and monkey brains [11], which was in agreement with the known anatomical distribution of AMPA receptors [12]. In addition, PET imaging using [^11^C]HMS011 showed moderate uptake in the rat and monkey brains and the distribution consistent with the results of the autoradiography. Furthermore, pre-administration of unlabeled HMS011 successfully blocked the uptake of radioactivity in a dose-dependent manner, supporting the specific binding of [^11^C]HMS011 in the rat and monkey brains. These data suggested that [^11^C]HMS011 has promising properties to allow in vivo imaging of AMPA receptors of the human brain [8, 11]. In the present study, we conducted the first investigative study of the safety and kinetics of [^11^C]HMS011 as a PET ligand for AMPA receptors in the human brain.

## Methods

### Radiopharmaceutical preparation

[^11^C]HMS011 was prepared by previously reported procedures [11]. In brief, [^11^C]CH3OTf gas was introduced to 2-(1-(3-aminophenyl)-2-oxo-5-(pyrimidin-2-yl)-1,2-dihydropyri-din-3-yl) benzonitrile (0.50 mg, 1.4 μmol) in dry acetone (250 μL) via a bubbling tube until radioactivity reached saturation, and the reaction mixture was subsequently dried at 80 °C under a N_2_ stream. The residue was dissolved in high-performance liquid chromatography (HPLC) eluent (1 mL) and purified by HPLC (Capcell Pak C18) using a mobile phase of CH_3_CN/H_2_O/TEA (4.5:5.5:0.01, *v*/*v*/*v*) at a flow rate of 5.0 mL/min to give [^11^C]HMS011. The injected radioactivity of [^11^C]HMS011 was 272.3–381.8 MBq [344.1 ± 42.5 MBq (mean ± S.D.)], and the molar radioactivity of the radioligand at the time of injection was 72.6–197.7 GBq/μmol [143.1 ± 49.2 GBq/μmol (mean ± S.D.)]. The injected mass was 1.9–3.7 nmol [2.7 ± 0.8 nmol (mean ± S.D.)].

### Subjects

Four healthy male subjects (NC1–4) [range, 22–24 years; mean ± S.D., 23.0 ± 1.0 years] participated in this study. Subjects with current or past psychiatric disorders, substance abuse, or organic brain disease were excluded based on their medical history and magnetic resonance (MR) imaging of the brain. Subjects also underwent a physical examination and blood and urine analysis to exclude physical illnesses. In order to evaluate the safety of [^11^C]HMS011, blood tests including a complete blood cell count and serum chemistry were conducted before and 2 h after injection of [^11^C]HMS011. The study protocol was approved by the Institutional Review Board of the National Institute of Radiological Sciences, Chiba, Japan. Written informed consent was obtained from each subject after complete explanation of the study. The current study was registered with the University Hospital Medical Information Network Clinical Trials Registry (UMIN000018938).

### Measurement of [^11^C]HMS011 in plasma

To obtain arterial input functions, arterial blood samples were taken manually 35 times after injection of [^11^C]HMS011 by the following schedule: at 10-s intervals to 2 min; at 30-s intervals to 3 min; at 1-min intervals to 10 min; at 12, 15, 20, 25, and 30 min; and at 10-min intervals to 120 min after injection. Due to technical problems, arterial blood sampling could not be performed in one subject (NC3). Thus, full kinetic analyses were performed for the other three subjects. Each blood sample was centrifuged to obtain plasma and blood cell fractions, and the concentrations of radioactivity in the whole blood and plasma were measured. The plasma-free fraction was measured by ultrafiltration (Centrifree, Merck Millipore, Billerica, MA).

The fractions of the parent and its radiometabolites in the plasma were determined by HPLC from eight samples in each subject (collected at 3, 10, 20, 30, 50, 70, 90, and 120 min after radioligand injection). The supernatant of the centrifuged samples was measured by radio-HPLC analysis (Waters XBridge OST C18 2.5 mm (10 × 50 mm). Acetonitrile (90%; A) and ammonium acetate (0.02 M; B) were used as mobile phases (40/60 A/B) at a flow rate of 5.0 mL/min.

### PET and MR scan procedures

After intravenous rapid bolus injection of [^11^C]HMS011, three-dimensional dynamic images were acquired in a PET camera for 120 min with 39 frames of increasing duration from 10 s to 5 min (10 s × 6, 20 s × 3, 1 min × 5, 3 min × 4, and 5 min × 20). All PET studies were performed with a Biograph mCT flow system (Siemens Healthcare, Erlangen, Germany), which provides 109 sections with an axial field of view of 16.2 cm. The intrinsic spatial resolution was 5.9 mm in-plane and 5.5 mm full-width at half-maximum axially. Images were reconstructed using a filtered back projection algorithm with a Hanning filter (6.0 mm full-width at half-maximum). All PET images were corrected for attenuation based on CT images, for random using the delayed coincidence counting method, and for scatter using the single-scatter simulation method. A head fixation device was used to minimize the subject’s head movement during the PET measurements.

MR images were acquired with a 3-T scanner, MAGNETOM Verio (Siemens Healthcare, Erlangen, Germany). Three-dimensional volumetric acquisition of a T1-weighted gradient echo sequence produced a gapless series of thin sagittal sections (TE = 1.95 ms, TR = 2300 ms, TI = 900 ms, flip angle = 9°, acquisition matrix = 256 × 256 × 250, voxel size = 1 × 1 × 1 mm). None of the subjects exhibited apparent structural abnormalities in MR images.

### Brain image processing

All PET images were spatially normalized to a standard anatomic orientation (MNI152 standard space; Montreal Neurological Institute, Montreal, QC, Canada) based on transformation parameters obtained from coregistered MR images. The automated anatomical labeling atlas implemented in PMOD version 3.6 was intersected with a binary gray matter mask created by a segmentation program of statistical parametric mapping (SPM8, Wellcome Trust Centre for Neuroimaging, London, UK) for the cortical regions. These volumes of interest were applied to the spatially normalized PET images to extract time-activity curves for the following regions: frontal cortex (219 cm^3^), cingulate cortex (17 cm^3^), parietal cortex (121 cm^3^), occipital cortex (97 cm^3^), temporal cortex (101 cm^3^), hippocampus (11 cm^3^), amygdala (3 cm^3^), caudate (12 cm^3^), putamen (12 cm^3^), pallidum (1 cm^3^), thalamus (7 cm^3^), cerebellum (133 cm^3^), pons (2 cm^3^), and centrum semiovale (5 cm^3^).

### Kinetic analysis

Single-input model: Regional total distribution volume (*V*_T_), an index of the receptor density that equals the ratio at equilibrium of the concentration of radioligand in tissue to that in plasma, was calculated with compartment models and graphical analysis using arterial input functions. The concentration of radioligand in tissue represents the sum of specific binding (receptor-bound) and non-displaceable uptake (sum of nonspecifically bound and free radioligand in tissue water). For compartment analysis, *V*_T_ was determined with one- and two-tissue compartment models using a radiometabolite-corrected plasma input function with the brain blood volume contribution fixed at 5%. An optimal compartment model was chosen on the basis of Akaike information criterion (AIC) [13], model selection criterion (MSC) [14], and goodness of fit assessed with *F* statistics [15]. For graphical analysis, the Logan plot [16] using a radiometabolite-corrected plasma input function with the brain blood volume contribution fixed at 5% was performed to estimate *V*_T_.

Dual-input model: Based on an initial analysis, we suspected that the radiometabolites of [^11^C]HMS011 may slowly enter the brain. We therefore used a dual-input graphical analysis to calculate *α* (slope of a regression in the graphical analysis, which equals the weighted sum of the total distribution volume of parent and radiometabolites), in which the effect of the metabolite on brain radioactivity was considered [8, 11].

1 $$ \frac{\int_0^t{C}_{\mathrm{b}}(t) dt}{C_{\mathrm{b}}(t)}=\alpha (t)\frac{\int_0^t{C}_{\mathrm{a}}^{\mathrm{P}+\mathrm{M}}(t) dt}{C_{\mathrm{b}}(t)}+\beta (t) $$

where ${C}_{\mathrm{a}}^{\mathrm{P}+\mathrm{M}}$ and *C*_b_ are the radioactivity concentrations of the total plasma and brain, respectively. In Eq. 1,

2 $$ \alpha =\left[1/\left(1+\delta \right)\right]{\alpha}^{\mathrm{P}}+\left[\delta /\left(1+\delta \right)\right]{\alpha}^{\mathrm{M}} $$

where *α* ^P^ and *α* ^M^ represent *V*_T_s of the parent and the radiometabolite, respectively, and *δ* is a plasma radiometabolite-to-parent concentration ratio at equilibrium.

Reference tissue model: To estimate the binding potential (BP*_ND_) of the radioligand in each brain region, we employed the original multilinear reference tissue model (MRTM_O_) [11]. Here, we distinguished BP*_ND_ from the original definition of *BP*_ND_ [13], since BP*_ND_ includes the additional contribution of radiometabolites entering the brain. Importantly, as shown in our previous study [14], BP*_ND_ is directly proportional to the target binding site density, *B*_avail_, in the situation where the radiometabolite enters the brain. Reference tissue models including MRTM_O_ employ time-activity data in a brain region devoid of specific binding components as input functions. Since our previous autoradiographic study demonstrated that there was minimum specific binding of [^11^C]HMS011 in the white matter of the monkey brains [15], we used the centrum semiovale as reference tissue in the current study. In addition to this region-of-interest-based MRTM_O_ analysis, we performed the voxel-based MRTM_O_ analysis to generate BP*_ND_ parametric images. All kinetic analyses were performed using the PMOD (version 3.6, PMOD Technologies Ltd., Zurich, Switzerland).

## Results

### Safety

There were no adverse effects of the [^11^C]HMS011 injection. The subjects did not complain of any subjective symptoms during or after the PET scans with [^11^C]HMS011. In addition, injection of [^11^C]HMS011 did not cause any changes in the blood tests or vital signs.

### Blood analysis

**Fig. 1:**
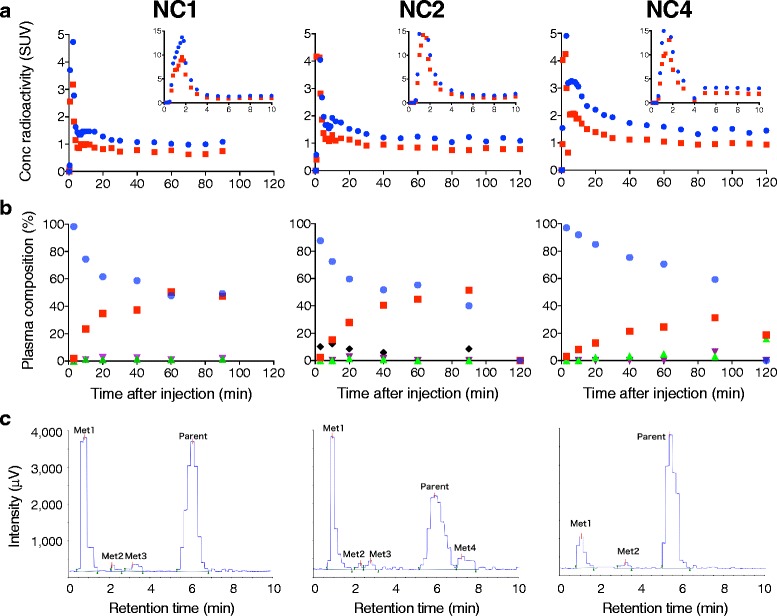
Concentration of radioactivity and composition of plasma radioactivity in arterial plasma after injection of [^11^C]HMS011 in three subjects (NC1, NC2, and NC4). **a** Concentration of radioactivity in plasma (*blue*) and whole blood (*red*). Values from 0 to 10 min and 10 to 120 min are shown in the two graphs with different *y*-axis ranges. **b** Composition of parent and radiometabolites in plasma. Parent (*blue*) and radiometabolites (metabolite 1: *red*, metabolite 2: *green*, metabolite 3: *purple*, and metabolite 4: *black*) are plotted against time after injection. Note that metabolite 4 was detected only in NC2. **c** Radiochromatogram of plasma at 20 min (NC1 and NC2) and 60 min (NC4) after injection of [^11^C]HMS011. Two or three hydrophilic radiometabolites (metabolites 1–3) were detected in all subjects. A lipophilic radiometabolite (Met4) was detected only in NC2

In plasma and whole blood, radioactivity showed a rapid increase and decline followed by a rather persistent presence after the administration (Fig. 1a). More than 50% of the radioactivity was unmetabolized [^11^C]HMS011 in the plasma at 60 min (Fig. 1b). Two or three radiometabolites of [^11^C]HMS011 appeared in the plasma of all the three subjects undergoing blood sampling immediately after the radioligand injection and were presumably more hydrophilic than the parent radioligand in light of their retention times in HPLC charts (Fig. 1c). One subject (NC2) showed a fourth radiometabolite very soon after the injection of [^11^C]HMS011. This radiometabolite was conceived to be more lipophilic than the parent radioligand, as it displayed a longer retention time in HPLC. The plasma-free fraction of [^11^C]HMS011 was 7.3 ± 0.2%.

### Brain uptake

**Fig. 2:**
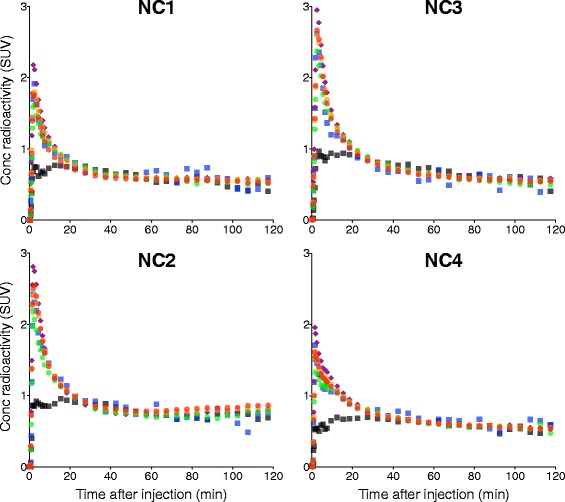
Time course of radioactivity in the frontal cortex (*red*), temporal cortex (*orange*), hippocampus (*green*), cerebellum (*purple*), pons (*blue*), and centrum semiovale (*black*) after injection of [^11^C]HMS011 in four subjects

**Fig. 3:**
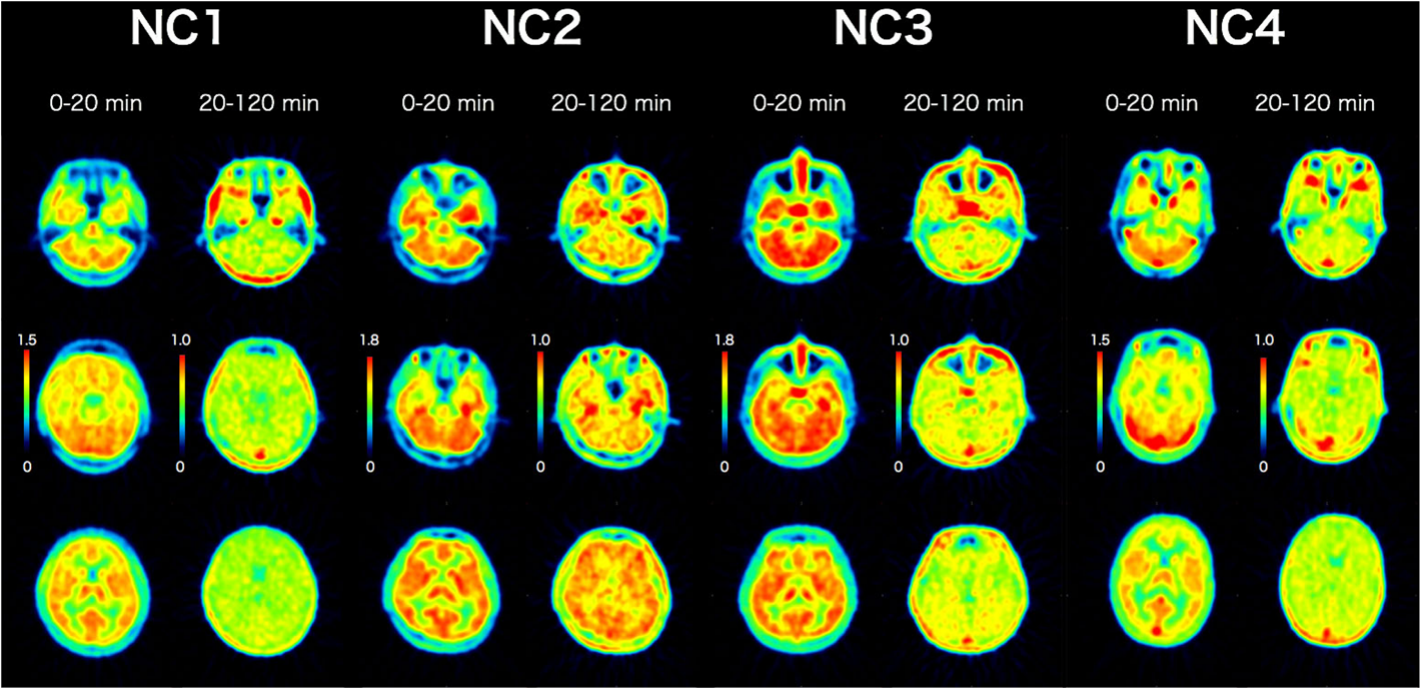
PET images of four healthy subjects after injection of [^11^C]HMS011. Horizontal PET images were obtained by averaging from 0 to 20 min and 20 to 120 min after injection of [^11^C]HMS011. Images were shown at the level of the cerebellum (*top*), pons (*middle*), and thalamus (*bottom*)

The peak brain uptake was observed within a few minutes after the injection of [^11^C]HMS011. The highest initial uptake was observed in the cerebellum with a standardized uptake value (SUV) of 2.0–2.9 (Fig. 2). This was followed by a fast washout of radioactivity in all the brain regions, resulting in regionally less variable radioactivity retention with SUV of 0.4–1.0 (Figs. 2 and 3). One subject producing a putative lipophilic radiometabolite (NC2) in the plasma showed higher radioactivity retention in the brain than the other individuals (Figs. 2 and 3).

### Brain kinetic analysis

**Fig. 4:**
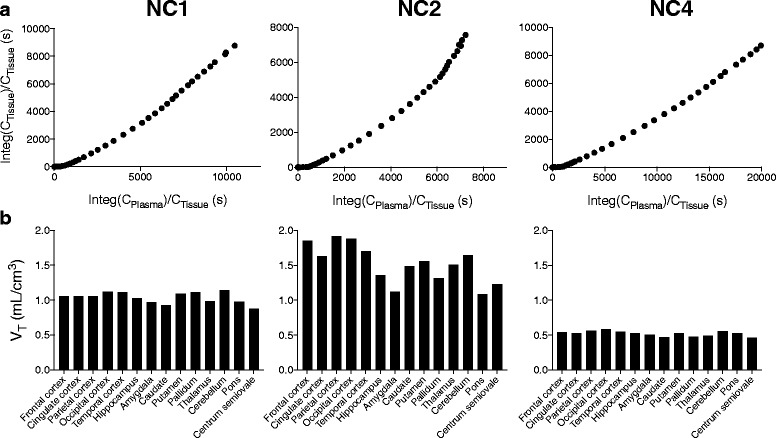
**a** Logan plots with parent input functions in the frontal cortex in three subjects. **b** Regional total distribution volumes (*V*_T_s) determined by Logan plots with parent input functions in three subjects

Kinetic analyses using parent plasma input functions revealed that the estimation of *V*_T_ was unstable and that the estimated *V*_T_ values were low throughout the brain. Using 120-min data collected from the three subjects, a two-tissue compartment model described the radioligand kinetics in the brain better than a one-tissue compartment model (Additional file 1: Figure S1). Indeed, AIC, MSC, and *F* test indicated that the two-tissue model fit (AIC/MSC 51.5 ± 46.7/3.6 ± 1.3) was superior to the one-tissue model fit (AIC/MSC 107.4 ± 14.5/2.1 ± 0.3) in all regions of the three subjects (*p* < 0.001 in *F* test for all regions). However, estimation of *V*_T_ values by the two-tissue compartment model was rather unstable in two of the subjects, as the regional mean of %SE in the fit for NC2 (33.8 ± 21.9%) and NC4 (26.6 ± 46.9%) was notably larger than that of NC1 (3.2 ± 1.1%). Logan graphical plots showed a linearity after 50 min in two subjects (NC1 and NC4) but a slight increase of the slope over time in one subject with a possibly lipophilic plasma metabolite (NC2) (Fig. 4a). There was a large variation in the *V*_T_ values estimated with the Logan graphical analysis among the three subjects (Fig. 4b). A significant main effect of subjects was revealed on the *V*_T_ values by analysis of variance (ANOVA) (*F* = 173.4, *df* = 2.13, *p* < 0.0001). *V*_T_ values were ~ 1 and ~ 0.5 in NC1 and NC4, respectively, and were almost uniform among regions. However, NC2 showed higher values (~ 1.0–1.5) with a larger regional variability.

**Fig. 5:**
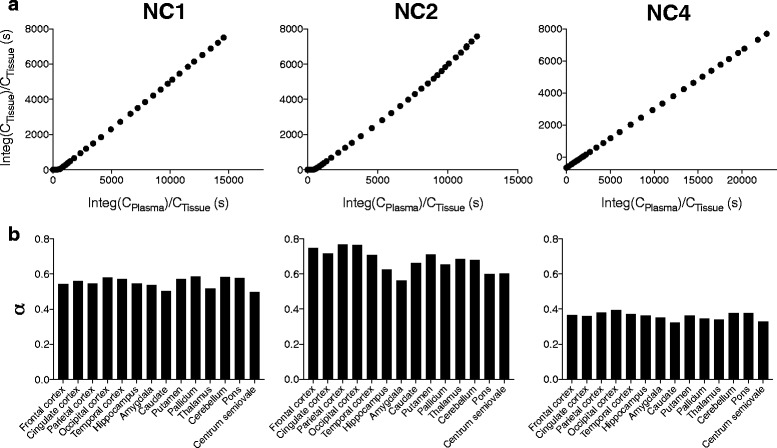
**a** Dual-input graphical plots in the frontal cortex in three subjects. **b** Regional distribution volumes (*α*) determined by the dual-input graphical model in three subjects

As a slight increase of the slope in the Logan graphical plots was observed in NC2, we suspected that lipophilic radiometabolites might slowly enter the brain. A dual-input graphical analysis was accordingly performed to estimate the distribution volume (*α*) in consideration of radiometabolites entering the brain. Using this model, the graphical plots were almost linear in all the three subjects (Fig. 5a), and average *α* values were less variable (0.6 ± 0.0, 0.7 ± 0.1, and 0.4 ± 0.0 for NC1, NC2, and NC3, respectively, Fig. 5b) than *V*_T_ values estimated by Logan graphical plots using parent-only input functions. A significant main effect of subjects was revealed on the *α* values by ANOVA (*F* = 284, *df* = 2.13, *p* < 0.0001).

**Fig. 6:**
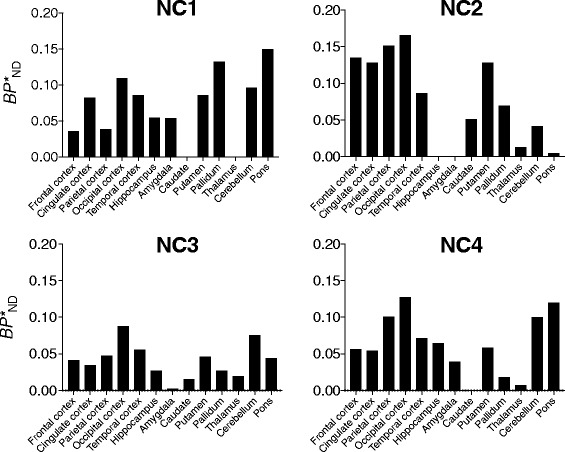
Regional binding potential values (BP*_ND_) estimated by region-of-interest-based MRTM_O_ with centrum semiovale as reference tissue in four subjects

**Fig. 7:**
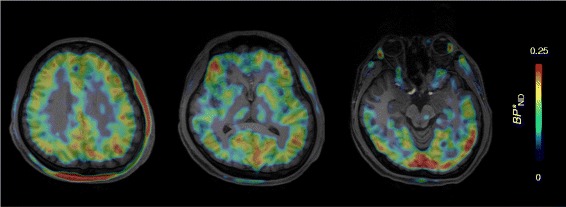
Parametric images of BP*_ND_ of the subject with lipophilic radiometabolites (NC2) created by voxel-based MRTM_O_ using centrum semiovale as reference tissue

Finally, a reference tissue model showed small radioligand BP*_ND_ values (BP*_ND_ = 0–0.15) in several cortical regions (Fig. 6). Accordingly, parametric BP*_ND_ images showed a dense distribution of voxels throughout gray matter, indicating specific binding of [^11^C]HMS011 in this area (Fig. 7). However, BP*_ND_ values were very small (< 0.25) (Fig. 7) without any significant main effect of subjects on *BP* ^*^_ND_ values in ANOVA (*F* = 1.476, *df* = 3.12, *p* = 0.251). These results impede accurate assessments of regional differences.

## Discussion

In this study, we detected specific binding signals of [^11^C]HMS011 in the human brain. [^11^C]HMS011 showed rapid brain uptake and clearance. Kinetic analyses indicated the possible entry of radiometabolites into the brain, because dual-input graphical analysis considering radiometabolites in the brain estimated *V*_T_ more reliably than compartment and Logan graphical analyses using parent-only input functions. A reference tissue model showed positive binding potential values, indicating the existence of specific binding in the human gray matter. However, the small binding potential values would hamper the possibility of using this radioligand to assess AMPA receptor density or occupancy.

In our plasma radiometabolite analysis, four radiometabolites of [^11^C]HMS011 were identified by HPLC. Among them, three were more hydrophilic than the parent radioligand and therefore considered to have only slight ability to enter the brain. In contrast, the fourth metabolite, which was observed only in one subject (NC2), was more lipophilic than the parent compound. In this subject, regional differences in *V*_T_ values were more prominent than in the other subjects, and Logan plot with a parent input function showed a slight but definite deviation from a straight line at later time points. These observations suggested that the lipophilic radiometabolite entered the brain gradually. Consistent with this interpretation, a dual-input graphical analysis yielded a linear plot even at the later time points in this subject.

This lipophilic radiometabolite appeared to be unique in humans, since only hydrophilic metabolites were observed in our previous investigation in rats and monkeys. This means that trying to identify lipophilic radiometabolites using plasma samples obtained from rats and monkeys would be difficult. We therefore performed a predictive analysis of the metabolism of [^11^C]HMS011 by a simulation (ADMET Predictor version 7.2, Simulations Plus, Lancaster, CA, USA). The result revealed a possible structure of the fourth metabolite ([^11^C]Metabolite-4), which could be produced by an additional methylation of the amino group and is more lipophilic than the parent compound (Additional file 1: Figure S2). Then, we synthesized [^11^C]Metabolite-4 and evaluated its kinetics in the rat brains. Contrary to our prediction, in vivo kinetics showed that brain uptake of [^11^C]Metabolite-4 was small (Additional file 1: Figure S3) and no retention was observed in rats (Additional file 1: Figure S4). Furthermore, HPLC analysis showed that the retention time of [^11^C]Metabolite-4 was longer than that of the lipophilic radiometabolite identified in the human subject. Consequently, the current experimental investigation could not provide any conclusive information on the chemical structure and kinetics of the lipophilic radiometabolite identified in the human study. However, it may give a clue to the development of a more suitable AMPA receptor PET ligand with high brain uptake and retention through the examination of this metabolite. Although exact mechanisms underlying variation of [^11^C]HMS011 metabolism among subjects remain unknown, it is likely related to individual variation of activity of hepatic cytochrome system that is involved in the metabolism of this drug. While the presence of such a radiometabolite is a complication, it does not necessarily preclude the quantification of radiotracer retentions, if the radiometabolite distributes uniformly in the brain in a non-specific manner.

To quantify the binding of [^11^C]HMS011, we chose MRTM_O_ because it offers stable estimation of a relatively low radioligand binding [16] and is applicable to a radioligand with its radiometabolites entering the brain. The BP*_ND_ values determined by MRTM_O_ are directly proportional to the target binding despite radiometabolites entering the brain [17]. In fact, we found small BP*_ND_ values in the cerebral cortical regions, indicating the specific binding components for [^11^C]HMS011 in the human brain. These results indicated that reference tissue models using the white matter as reference tissue can be useful for quantification of AMPA receptors; however, this methodology will need to be validated using PET ligands for AMPA receptors with higher amounts of specific binding than [^11^C]HMS011.

The reasons for the small amount of specific binding of [^11^C]HMS011 in humans are still unknown. The brain uptake of [^11^C]HMS011 was similar between monkeys and humans (peak SUV ~2), while the amount of specific binding appeared to be higher in monkeys than in humans [18]. Actually, [^11^C]HMS011 had a moderate affinity for human AMPA receptors with *K*_D_ values approximating 15 nM (Additional file 1 and Additional file 1: Figure S5). These values were slightly larger than the *K*_i_ value of HMS011 in the rat brain (10 nM) in our previous assay [11], partly explaining the low specific radioligand binding in the current human PET study.

We found a retention of [^11^C]HMS011 in the human brainstem similar to that in the other regions, in contrast to the low radioligand retention in the brainstem of rats and monkeys [11]. Because mRNA levels are low in this region for all subunits of AMPA receptors, this retention in the human brainstem can reflect the binding of [^11^C]HMS011 to different molecules such as kainate receptors [12], adenosine 2A (A2A) receptors, and 18-kDa translocator protein (TSPO) [11]. Of these, binding to A2A receptors does not explain the current findings, as they are expressed more prominently in the striatum than in the brainstem in both rats and humans [19, 20]. Meanwhile, TSPO might be the other target molecule of [^11^C]HMS011, as the amount of binding sites in the brainstem is greater in humans than in rats.

The small sample size is a limitation of the current study, and any generalization of our findings needs to be approached with caution. Notwithstanding this limitation, our results provide information that is helpful for the future development of a reliable PET ligand for human AMPA receptors.

## Conclusions

Human PET study using [^11^C]HMS011 showed some specific binding in the cerebral cortex. However, [^11^C]HMS011 may not be ideal for practical AMPA receptor PET imaging because of the small magnitudes of detectable specific binding signals. Our results suggest the needs for further development of radioligands that have more suitable properties for in vivo AMPA receptor imaging.

## Additional file

Additional file 1: Supplementary m﻿aterials including methods and results of a receptor binding assay to determine the affinity of [^11^C]HMS011 for human AMPA receptors and supplementary figures. (PDF 2639 kb)

## Abbreviations

[^11^C]HMS011: 2-(1-(3-([^11^C]methylamino)phenyl)-2-oxo-5-(pyrimidin-2-yl)-1,2-dihydropyridin-3-yl) benzonitrile
AIC: Akaike information criterion
AMPA: α-Amino-3-hydroxy-5-methyl-4-isoxazole propionate
*BP**_ND_: Binding potential
HPLC: High-performance liquid chromatography
MR: Magnetic resonance
MRTM_O_: The original multilinear reference tissue model
MSC: Model selection criterion
NBQX: 2,3-Dihydroxy-6-nitro-7-sulfamoyl-benzo(f)quinoxaline
NMDA: *N*-methyl-d-aspartate
PET: Positron emission tomography
S.D.: Standard deviation
TSPO: 18-kDa translocator protein
*V*_T_: Total distribution volume
