# Supplementary material for: A human PET study of [11C]HMS011, a potential radioligand for AMPA receptors
Source: EJNMMI Res. 2017 Aug 16;7:63. doi: 10.1186/s13550-017-0313-0 (PMC5559406; doi:10.1186/s13550-017-0313-0)
Supplement: Additional file 1: — Supplementary m﻿aterials including methods and results of a receptor binding assay to determine the affinity of [11C]HMS011 for human AMPA receptors and supplementary figures. (PDF 2639 kb) [file 13550_2017_313_MOESM1_ESM.pdf]

Additional file

Receptor Binding assay

We performed a binding assay using human brain homogenates in order to determine the affinity of [^{11}C]HMS011 for human AMPA receptors.

Methods: Post-mortem human brains were obtained from autopsies carried out at the Fukushimura Hospital. Frozen tissues derived from the frontal cortex of a healthy subject were homogenized in 50mM Tris-HCl buffer, pH 7.4, containing protease inhibitor cocktail (cOmplete^{TM}, EDTA-free; Roche), and stored at -80° pending analyses. To evaluate the radioligand binding and its homologous blockade, these homogenates (200mg tissue) were incubated with 5 nM [^{11}C]HMS011 in the absence or presence of unlabeled HMS011 at varying concentrations ranging from 10^{-11} to 10^{-6} M in Tris-HCl buffer, pH 7.4, for 30 min at room temperature. These radioligand concentrations were determined in consideration of the radioactive half-life of ^{11}C (approximately 20 min). Non-specific binding of [^{11}C]HMS011 was determined in the presence of 5 x 10^{-7} M HMS011. Samples were run in quadruplicates, and specific radioligand binding was determined as pmol/g tissue. Inhibition constant (K_i) and percentage of displacement were determined by using a non-linear regression fit to a concentration-binding plot based on one-site and two-site binding models derived from the Cheng-Prusoff equation with GraphPad Prism version 5.0 (GraphPad Software), followed by F-test for the model selection. In a one-site homologous blockade model,

dissociation constant (K_D) and maximum number of binding sites (B_{max}) were calculated using the following equations:

$$K_D = K_i = IC_{50} - [Radioligand] \qquad (1)$$

$$B_{max} = \frac{Top-Bottom}{[Radioligand]/(K_D+[Radioligand])} \qquad (2)$$

where IC_{50} and [Radioligand] are concentration of the competitor inducing 50% inhibition and radioligand concentration, respectively, and Top and Bottom are upper and lower plateaus of the plot curve, respectively.

Results:

Total (specific + non-specific) radioligand binding and its blockade are depicted in Figure S5. Specific [^{11}C]HMS011 binding in the human frontal cortex tissue was homologously inhibited by non-labelled HMS011 in a concentration-dependent fashion, and the blocking curve was demonstrated by a one-site model, indicating the presence of binding sites for [^{11}C]HMS011 with 15.34 nM of K_i (= K_D), which was slightly larger than the K_i of this compound in rat brain samples [1]. B_{max} value was estimated to be 18.82 pmol/g. Based on these results obtained by binding assay and other parameters (f_p=0.073 and V_{ND}=0.87), BP_{ND} value of [^{11}C]HMS011 to human AMPA receptors is estimated to be about 0.1. This BP_{ND} value is similar to those calculated by $MRTM_O$ in the present PET study, supporting the low-level specific radioligand binding.

References

1. Oi N, Tokunaga M, Suzuki M, Nagai Y, Nakatani Y, Yamamoto N, et al. Development of Novel PET Probes for Central 2-Amino-3-(3-hydroxy-5-methyl-4-isoxazolyl)propionic Acid Receptors. J. Med. Chem. 2015;58:8444–62.

Additional file

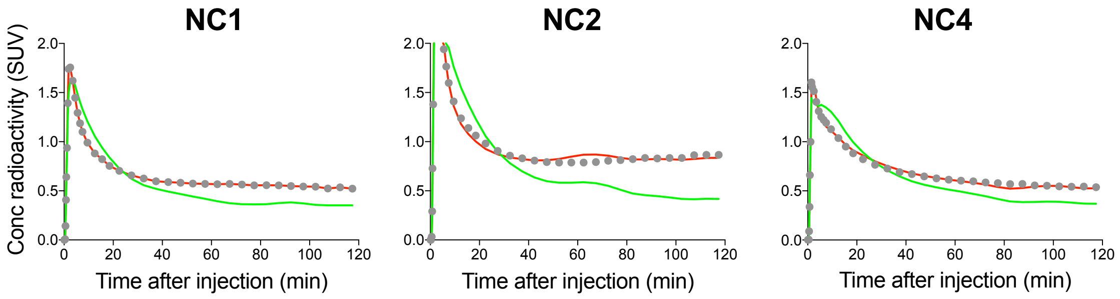

Figure S1 Fitting curves of one-tissue compartment model (green lines) and two-tissue compartment model (red lines) on time-activity curves in the frontal cortex (filled circles). Two-tissue compartment model provided better fitting than one-tissue compartment model for all 3 subjects.

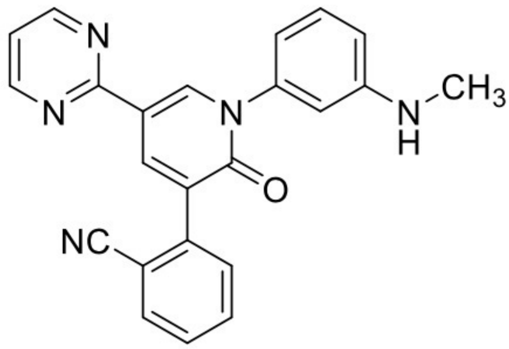

HMS011

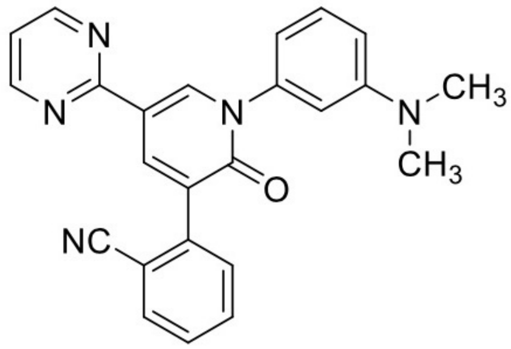

Metabolite-4

Figure S2 A chemical structure of HMS011 and a proposed chemical structure of the fourth metabolite.

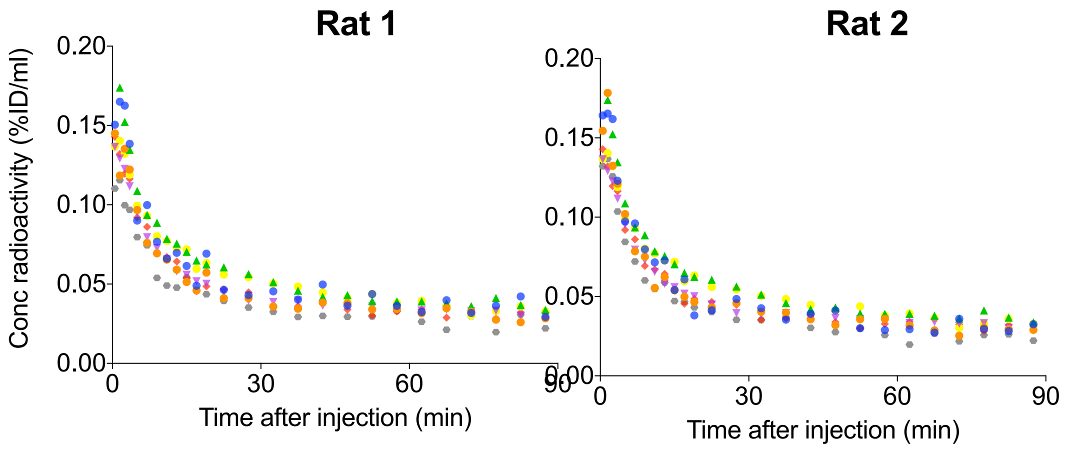

Figure S3 Average time-course of radioactivity in medial prefrontal cortex (blue), striatum (orange), cerebellum (green), hippocampus (purple), neocortex (yellow), thalamus (red), and brain stem (gray) after injection of [^{11}C]Metabolite-4 in two wild-type rats.

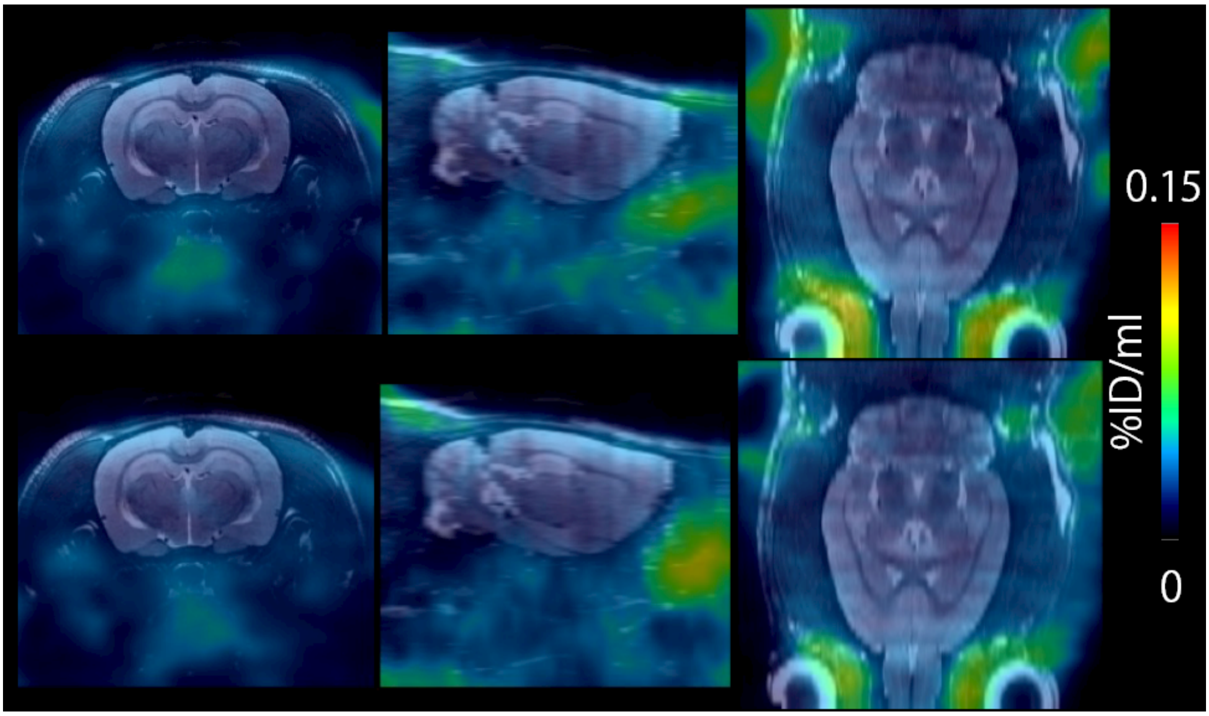

Figure S4. PET images (0–90 min) and time activity curves of [^{11}C]Metabolite-4 in two wild-type rats (weight: 636 and 557 g and dose: 168.6 and 167.9 MBq for rats on the top and bottom rows, respectively).

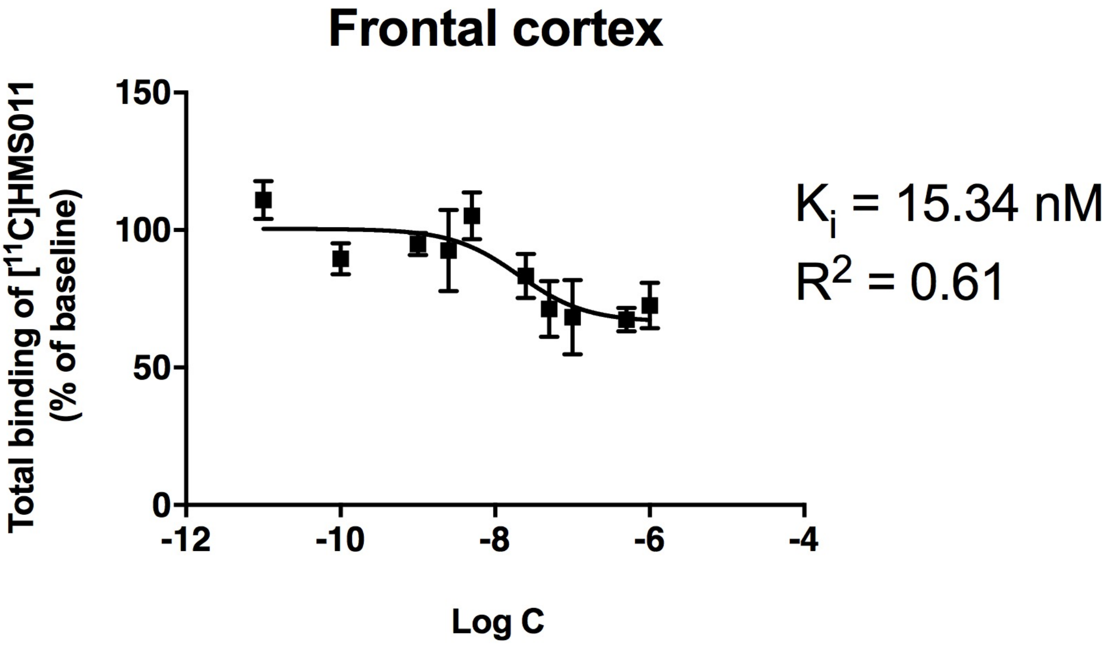

Figure S5. Binding of [^{11}C]HMS011 in homogenates derived from the human frontal cortex. The specific binding of [^{11}C]HMS011 was blocked by non-labeled HMS011. This homologous blockade was described by a one-site model, and parameters resulting from a curve fit are indicated in the graph. Data are presented as mean ± standard error.
